# Effects of Ventilation Improvement on Measured and Perceived Indoor Air Quality in a School Building with a Hybrid Ventilation System

**DOI:** 10.3390/ijerph15071414

**Published:** 2018-07-05

**Authors:** Camilla Vornanen-Winqvist, Heidi Salonen, Kati Järvi, Maria A. Andersson, Raimo Mikkola, Tamás Marik, László Kredics, Jarek Kurnitski

**Affiliations:** 1 Department of Civil Engineering, Aalto University, Rakentajanaukio 4, 02150 Espoo, Finland; heidi.salonen@aalto.fi (H.S.); kati.jarvi@aalto.fi (K.J.); maria.a.andersson@helsinki.fi (M.A.A.); raimo.mikkola@aalto.fi (R.M.); jarek.kurnitski@aalto.fi (J.K.); 2 Department of Microbiology, University of Szeged, Közép fasor 52, H-6726 Szeged, Hungary; mariktamas88@gmail.com (T.M.); kredics@bio.u-szeged.hu (L.K.); 3 Department of Civil Engineering and Architecture, Tallinn University of Technology, Ehitajate tee 5, 19086 Tallinn, Estonia

**Keywords:** ventilation, hybrid ventilation, indoor air quality, mycobiota, indoor air questionnaire, school building, *Trichoderma citrinoviride*

## Abstract

Ventilation system design and operation may significantly affect indoor air quality (IAQ). The aims of this case study were to investigate the functionality of a supply air fan-assisted hybrid ventilation system in a newly built school building with reported IAQ problems and to determine the effects of ventilation improvement on measured and perceived IAQ. The ventilation system function was researched simultaneously with IAQ measurements, with an analysis of total volatile organic compounds (TVOC), single volatile organic compounds (VOCs), and indoor mycobiota, and with questionnaires about perceived IAQ. At the baseline, an operational error of the ventilation system was found, which prevented the air from coming into the classrooms, except for short periods of high carbon dioxide (CO_2_) concentrations. After the ventilation operation was improved, a significant change in indoor mycobiota was found; the dominant, opportunistic human pathogenic species *Trichoderma citrinoviride* found in settled dust in the classroom before the improvement was no longer detected. In addition, the concentrations of CO_2_, TVOC, and some single VOCs, especially toluene and decamethylcyclopentasiloxane, decreased. The analysis of the questionnaire results indicated that the perceptions of unpleasant odors and stuffy air decreased, although a statistically significant improvement in perceived IAQ was not observed. The results provided evidence that the properly controlled hybrid ventilation system operating in mechanical supply mode provided adequate ventilation and was effective in decreasing the concentrations of some indoor-generated pollutants. With simple ventilation adjustments, microbiological exposure from building structures might be prevented.

## 1. Introduction

In Finland, moisture damage and ventilation disadvantages are the most common problems as they are reported in more than 50% of school buildings [1]. A recent Finnish study found that 58% of Finnish schools suffer from insufficient ventilation [2]. School environments are often complex and involve several interconnected factors that affect the health of occupants [3,4,5]. Current evidence shows that classroom conditions are significantly associated with the respiratory symptoms of teachers [6]. Kielb et al. [7] found that one or more perceived symptoms were most strongly associated with reported dust and dust reservoirs, mold and moldy odors, and paint odors. Symptoms of sick building syndrome (SBS) were associated with perceptions of stuffy air, dry air, and electricity [8]. Further, teachers’ perceptions of neuro-physiological symptoms, e.g., headache, fatigue, and difficulties in concentrating, were significantly increased with every 100 ppm increase in maximum classroom CO_2_ concentrations [9].

According to epidemiological studies, in general, higher ventilation rates (up to 25–40 L/s per person) reduce negative health outcomes, and with minimum rates of ventilation (above 6–7 L/s), some (mainly acute) health outcomes can be avoided [10]. In a review study, Sundell et al. [11] reported that lower ventilation rates might increase the incidence of respiratory infections, asthmatic symptoms, inflammation, and short-term sick leave. Correspondingly, teachers working at schools with good perceived IAQ have decreased risk for short-term sick leave (one to three days) [12]. In addition, it is well documented that both thermal conditions and IAQ affect the performance of students [13].

Cellulolytic fungi that require high water content to survive, such as *Trichoderma* species, are well adapted to colonize water-damaged buildings. Members of this genus are often found on wet manufactured wood and gypsum boards from schools and public buildings [14,15]. Building materials contaminated with *Trichoderma* species emit high amounts of conidia into the indoor air [15]. Conidia and hyphal fragments containing toxic peptaibols have been shown to provoke histamine release and disrupt the membranes of exposed target cells [15,16]. Exposure to viable conidia emitted from potentially pathogenic *Trichoderma* species, such as *T. longibrachiatum* and *T. citrinoviride*, represents an additional health risk [14,17]. Measurement of cultivable conidia from pathogenic and toxigenic fungi in settled dust in schools is an easy method to determine the potential health risk associated with changes in ventilation and fluctuations in indoor air pressure [18]. House dust is a long-term reservoir of indoor fungi, therefore it is frequently collected to evaluate cumulative exposure and fungal composition [19,20,21].

Ventilation plays a major role in creating a healthy and pleasant indoor environment, especially in modern airtight buildings. Hybrid ventilation systems are developed to combine the benefits of natural and mechanical ventilation, in order to increase the use of sustainable technologies, reduce energy consumption and to create healthy, comfortable indoor environment [22,23]. Natural ventilation is often well adopted by the occupants but may be inadequate in some climates and buildings. In the case of hybrid ventilation, mechanical components compensate the shortcomings of natural ventilation. Many comparative studies of natural, mechanical and hybrid ventilation systems have been published from the aspects of energy efficiency, thermal conditions, CO_2_ levels, and system simulations [24,25,26]. However, there are no studies available about reported IAQ problems and microbiological exposure in school buildings with hybrid ventilation systems.

In Helsinki, Finland, only a few public school buildings have hybrid ventilation systems. This study was conducted as part of the Finnish “EURA” and “TOXICPM” research projects (see acknowledgements) concerning IAQ and ventilation in new and renovated school buildings and microbial toxin transport mechanisms.

The aim of our study was to investigate the functionality of a hybrid ventilation system in a newly built school building with reported IAQ problems and to determine the effects of ventilation system improvement on measured and perceived IAQ. The effects of simple but essential improvement in the ventilation system were studied most of all from the point of approvable IAQ. The occurrence of indoor mycobiota before and after the improvements was investigated from an encompassing long-term dust sample. To our knowledge, except from our earlier intervention study of ventilation positive pressure [18], no comparative studies have been published before.

## 2. Materials and Methods

### 2.1. Building Characteristics

The study was conducted in the building of a comprehensive school in Helsinki, Southern Finland, where children from the ages of 7 to 16 were educated. Approximately 700 students and 70 staff members worked in the school. It was built in 2009, and the owner of the building (City of Helsinki, Urban Environment Division, Buildings and Public Areas, Built Assets Management; later in the paper called as the Built Assets Management of City of Helsinki), had experienced difficulties in maintaining sufficient ventilation and good IAQ in the building. Since 2010, several IAQ-related investigations and repairs had been conducted. For example, according to numerous investigation reports and information from the Built Assets Management of City of Helsinki, flooring was replaced because the concrete slabs were moist, local moisture damage was repaired, ventilation was adjusted, and air leaks were sealed. At the time of this research, the occupants had reported severe IAQ-related symptoms and discomfort during the past few years in different sections of the building, especially in the one under study. 

The building featured two separate ventilation systems: mechanical supply and extract ventilation with heat recovery in one section of the building, and fan-assisted natural ventilation (hybrid ventilation) in two identical sections. One section consisted of two floors. However, it should be noted that the building sections with different ventilation systems were not completely separated. Air was mixed between the sections since the main entrance of the building is located in the mechanically ventilated section, which has to be crossed in order to reach the hybrid-ventilated sections.

**Figure 1:**
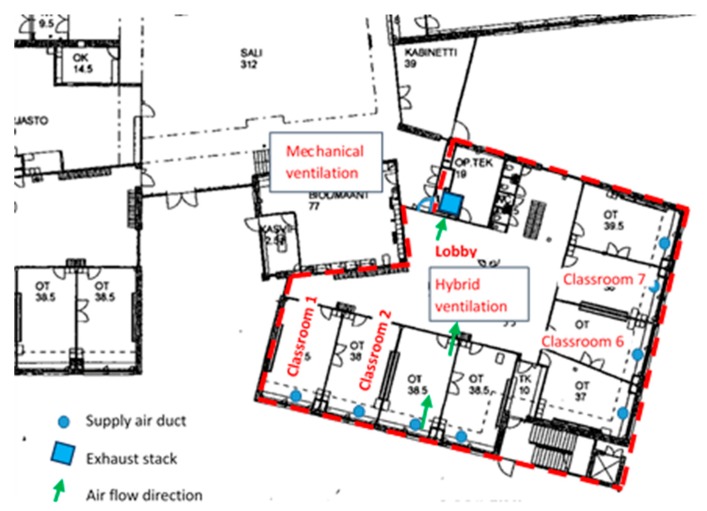
The studied building section (first floor) and locations of the supply air ducts and exhaust stack. Air flow direction is shown by arrows as an example in one classroom. Measurements were conducted mainly in Classrooms 1 and 2.

This study focused on one hybrid-ventilated section of the building, which consisted of two equal areas in the first and second floor, was served by one air handling unit and occupied by approximately 30% of the school teachers and students. Occupants had reported the most severe symptoms and discomfort in the studied section, especially in the first floor. The studied section consisted of a first floor lobby surrounded by eight classrooms and toilets. The layout is presented in Figure 1.

Supply air was taken into the building section by a supply air tower, where it was also filtered and brought to an air handling unit located in the basement underneath the building section. Supply air (assisting) fan was designed for on-demand use, but due to the prolonged IAQ problems, it was running constantly at full speed, designed for circumstances of CO_2_ level exceeding 1200 ppm. The designed supply air rate was 8 L/s/person, and altogether 2000 L/s for the entire building section (two floors).

**Figure 2:**
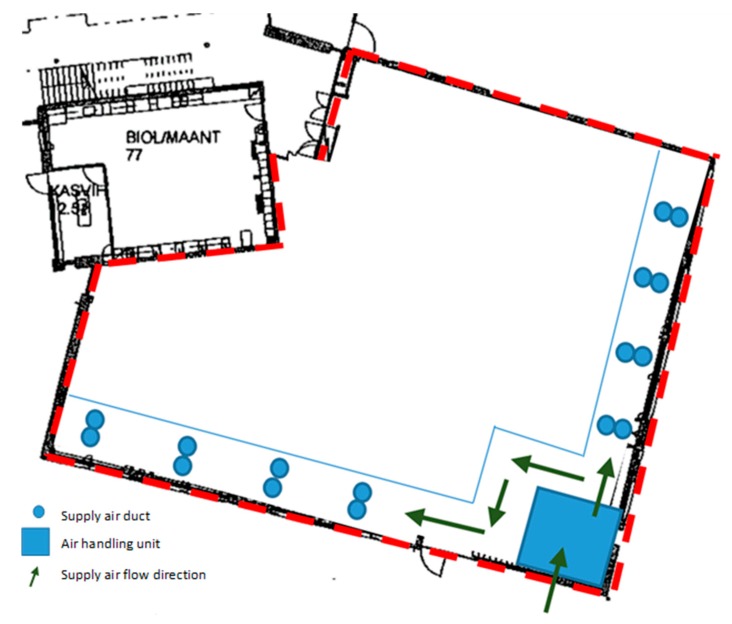
Location of the underground supply air chamber in relation to the studied building section (limited by a blue line): air handling unit, corridors, and terminal units of supply air ducts leading to the first- and second-floor classrooms. Direction of the supply air movement is marked with arrows.

After the air handling unit, supply air entered a chamber with two corridors, where it was heated with radiators. The cross-section area of the corridor space was 2 m^2^. Each classroom of the building section (altogether, 16 classrooms on two floors) had its own supply air duct beginning from these underground corridors. The layout of the supply air corridors and their location in relation to the section of the building under study are shown in Figure 2.

Classroom supply air rates were adjusted by motorized dampers located at the beginning of the supply air ducts. The dampers were designed to be a minimum of 20% open in basic situations to provide base ventilation for the classrooms. Classroom-specific carbon dioxide (CO_2_) sensors controlled the dampers after the CO_2_ level exceeded approximately 500 ppm in order to increase the supply air rate on demand. The supply air intake was controlled by an automatic remote control system.

**Figure 3:**
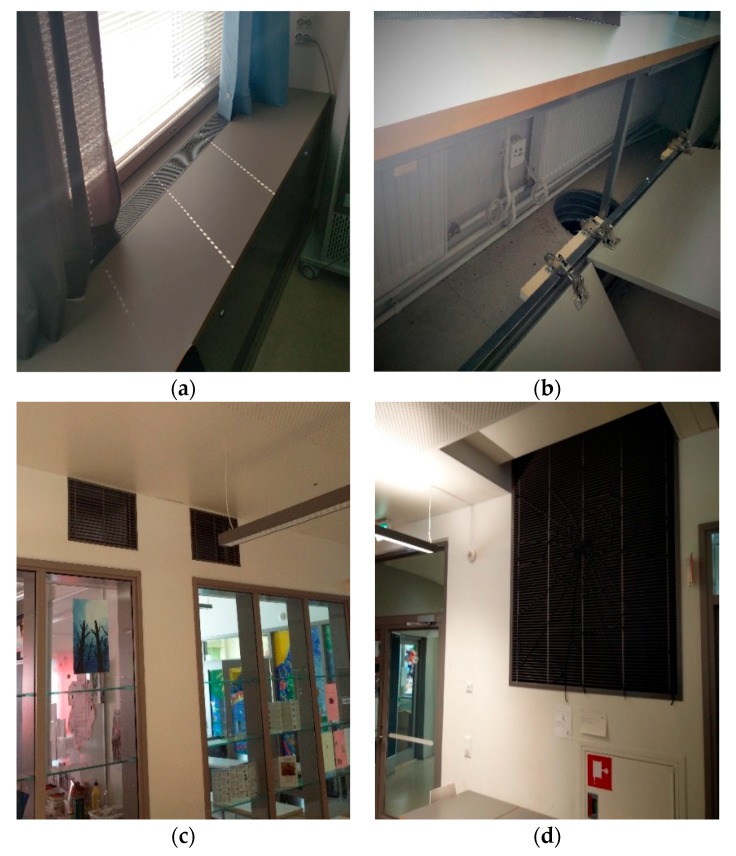
(**a**,**b**) Supply air grill and duct in the classrooms; (**c**) transfer air grilles in the partitions between classrooms and the lobby and (**d**) exhaust stack in the lobby.

Air was brought to the classrooms through grilles under the window sills, where the radiators were also located. From the classrooms, air was transferred to the lobby via grilles in the partitions, and then extracted outdoors from the lobby via a large exhaust stack, as presented in Figure 3.

### 2.2. Functionality of the Hybrid Ventilation System

According to the building’s management, occupants had reported significant discomfort, stuffy air, and unpleasant odors, especially in the section of the building under study. Bad odors and displeasing IAQ were easily observed during the researchers’ first visits to the building. The causes of occupants’ symptoms and discomfort were not identified, even after several investigations, but were suspected to be impurities that infiltrated the building through air leakages.

Ventilation function was investigated and reported previously [27] using pressure difference measurements, tests, and observations of the dampers in the supply air corridor with varied CO_2_ concentration; air flow measurements in the classrooms; and pressure difference measurements in the classrooms with varied door positions and damper settings.

The ventilation system was found to be severely malfunctioning, since the supply air dampers were completely closed due to a remote control system error that was defectively treated. Supply air was enabled to enter the ducts and classrooms only during short periods when CO_2_ concentration exceeded 500 ppm. Thus, the CO_2_ concentrations were sustained at an acceptable level, but the classrooms still had no base ventilation. There was a complete lack of supply air in the classrooms when they were unoccupied. According to a MERMAID-study, ventilation operation for slightly longer than the occupancy period seemed to be the best compromise for IAQ and energy consumption [28].

**Figure 4:**
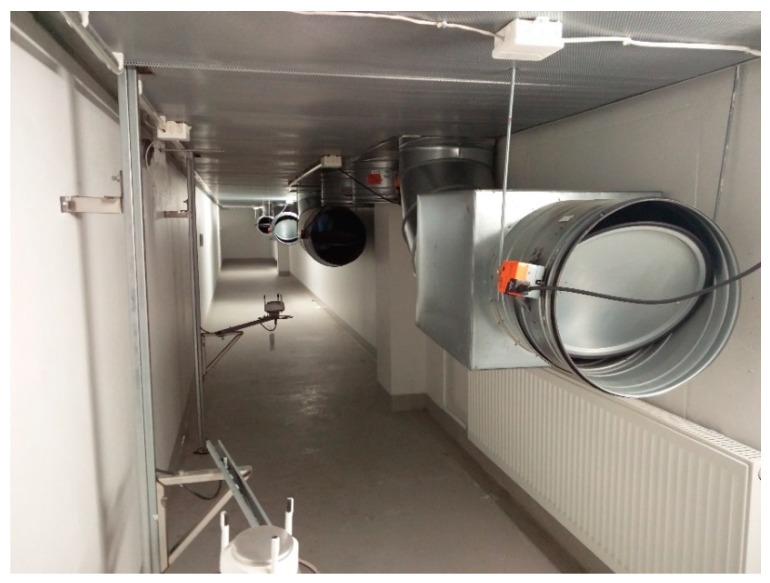
Supply air duct corridor and classroom-specific ducts’ terminal units in the basement. Dampers are opened 20%, as designed.

The underground supply air duct corridor and a duct damper that is opened as required for base ventilation are shown in Figure 4.

After initial investigations, the proper remote control system adjustments were carried out by a competent company, and base ventilation was provided to the classrooms after an approximately two-year break. 

Overall, the ventilation system was found to be complex and barely controllable [27]. The form of the supply air corridors (one straight and one angular) caused pressure loss and thus divided supply air unequally among the classrooms. Classrooms 1 and 2, the supply air ducts of which were located in the angular corridor and at the utmost from the air handling unit, seemed to have the least amount of supply air due to the pressure loss in the corridor. Furthermore, the classroom door positions affected the pressure relations throughout the whole building section. Thus, the hybrid ventilation system was shown to have some prerequisites for appropriate operation. Constant provision of the demanded supply air could be supported by some technical changes to the adjustments and control system.

### 2.3. Measurements in the School

In order to study the perceived and measured indoor air quality before (May 2016) and after (March 2017) the ventilation system improvements, a set of measurements were performed, mainly in Classrooms 1 and 2 where the occupants had reported the most severe symptoms and discomfort according to the Built Assets Management of City of Helsinki, and which were located most unfavorably in relation to the air handling unit. Classrooms 1 and 2 were next to each other, and could also be used as a combined space since there was a door in the partition. Additional measurements were conducted in the lobby and in some other classrooms in the section of the building under study. The measurement methods are presented in Table 1.

#### 2.3.1. Air Flow Rates and Pressure Differences across the Building Envelope

Air flow rates were measured as instant measurements before and after the improvement. Damper positions were controlled by increasing CO_2_ concentrations in the classrooms.

Pressure differences across the building envelope were measured in Classrooms 1 and 2 continuously for one week before, and two weeks after the ventilation improvement. A plastic tube with a copper core was placed outside by a window that was not normally open. A measurement device and logger were placed inside near the window.

#### 2.3.2. Indoor Air Quality (IAQ) Measurements

Temperature (T), relative humidity (RH), and CO_2_ concentrations were measured in Classrooms 1 and 2 for a one-week period before, and a two-week period after the ventilation improvement. Measurement devices were placed on the teacher’s desk in the front of the room, away from the teacher’s breathing zone when seated and as close to the horizontal central area of the room as possible.

Volatile organic compounds (VOCs) were measured in Classroom 2 before and after the ventilation improvement. VOC sampling and analysis were carried out according to the ISO 16000-6 standard [29]. Air samples were taken from the central area of an empty, closed room in the main working zone at a height of 1.5 m. Samples were collected in stainless steel tubes (Markes International Ltd., Llantrisant, UK) packed with Tenax TA (60/80 mesh) and Tenax TA-Carbograph 5TD using GilAir Plus air sampling pumps (Sensidyne, St. Petersburg, FL, USA) at a flow rate of 200 mL/min for 40 min. 

Before ventilation improvement, VOC-analyses were conducted at Aalto University, and after improvement, they were conducted at the Finnish Institute of Occupational Health (FIOH) due to reorganization of the project resources. In the Aalto University analysis, total volatile organic compounds (TVOCs) and single compounds with concentrations over 1 µg/m^3^ were analyzed, while in the FIOH analysis, concentrations less than 1 µg/m^3^ were also covered. At Aalto University, the samples were desorbed using a thermal desorption unit (TD-100, Markes International Ltd.) and analyzed using a gas chromatograph (Clarus 580, Perkin-Elmer Ltd., Beaconsfield, UK) equipped with a Clarus 600T (Perkin-Elmer Ltd.) mass selective detector. VOCs were quantified by the scan (50–400 *m*/*z*) mode. TVOC concentrations were determined from TVOC area (*n*-hexane to *n*-hexadecane) and calculated as toluene equivalents, and concentrations of the individual compounds were calculated either using pure reference compounds or as toluene equivalents. The concentrations of single compounds were also determined from the chromatogram before and after the TVOC area. In the case of such compounds, the quantitative results were as indicative.

Reference compounds (Indoor Air Standard, 50 components, 49148-U, Sigma-Aldrich, St. Louis, MO, USA) and the NIST 2011 Mass Spectral Library automated mass spectral deconvolution and identification system (AMDIS) were used for identification during the analysis at Aalto University, while at FIOH, where samples collected using Tenax TA-Carbograph 5TD steel tubes were analyzed, the Wiley database was also used. The detection limit was 0.2 µg/m^3^ (not included in sum concentration).

The formaldehyde concentration of indoor air was measured using an FM-801 formaldehyde meter (GrayWolf Sensing Solution, Sheldon, IA, USA). Fine particulate matter (PM_2.5_) was measured using a MIE pDR-1500 (Thermo Fisher Scientific, Franklin, MA, USA) nephelometer equipped with a PM_2.5_ size-selective inlet cyclone. Formaldehyde and PM_2.5_ were measured continuously for a one-week period before the ventilation in Classroom 2 was improved, in order to determine the indoor conditions while the ventilation system was dysfunctional. Measurement devices were placed in the back of the room at a height of 1.5 m and as close to the central area of the room as possible.

#### 2.3.3. Characterization of Mycobiota in Indoor Dust

Mycobiota in indoor dust was obtained from the settled dust collected from Classrooms 1 and 2 and the lobby before the ventilation improvement (May 2016) and 10 months after the improvement (March 2017). These mycobiota were characterized in three stages, as described previously [18]: sampling of dust, rapid toxicity screening of single colonies, and characterization and identification of the fungal isolates.

Dust samples were wiped into a clean plastic bag (Minigrip: Amerplast, Tampere, Finland) from ca. 30 × 30 cm^2^ surfaces 1–2 m above floor level. The dust (ca. 10 mg) was spread with a sterile cotton swab onto malt extract agar (MEA) plates (malt extract 15 g: Sharlab, Barcelona, Spain; agar 12 g: Amresco, Solon, OH, USA, in 500 mL of H_2_O). Culture plates were inoculated, sealed, and cultivated at 22 °C for four weeks.

For initial toxicity screening, 10–20 mg of biomass (wet weight) from each colony of the original culture plates was looped into 0.2 mL of ethanol and heated in a water bath for 10 min at 80 °C. The obtained ethanolic lysates were exposed to porcine spermatozoa and kidney tubular epithelial cells (PK-15, Finnish Food Safety Authority, EVIRA, Helsinki, Finland). The lysate was considered toxic when 2.5 vol % decreased boar sperm motility or 5 vol % decreased proliferation of PK-15 cells by >50% compared to the sham-exposed control. Boar sperm motility inhibition assay (BSMI) measuring motility inhibition (i.e., inability of resting sperm cells exposed for one day at room temperature to respond to induction of motility) was performed according to Andersson et al. [30]. The inhibition of cell proliferation (ICP) assay with PK-15 cells and determination of EC_50_ concentrations followed the methods described by Bencsik et al. [31]. Colonies that displayed toxicity were pure-cultured and identified to the genus or species level.

Fungal colonies were grouped into eight morphotypes based on their morphology on MEA, ability to grow at 37 °C, light microscopy results for conidia and conidiophores, and responses in the two toxicity assays, BSMI and ICP. The isolates were compared to the reference strains from the HAMBI culture collection or identified according to the process described by Samson et al. [32]. A representative of the morphotype of toxigenic *Trichoderma* able to grow at 37 °C was identified by the sequence analysis of the ribosomal RNA gene cluster’s internal transcribed spacer (ITS) region [33].

#### 2.3.4. Indoor Air Questionnaire

Indoor air-related symptoms and discomfort of the occupants were recorded with the standardized Indoor Air Questionnaire of FIOH twice during the research: in May 2016 (before the ventilation improvement) and in January 2017 (after 6 months of working in the building after the improvement). 

The questionnaire was based on the Örebro Indoor Climate Questionnaire (MM40) [34]. In the questionnaire, respondents were asked to recall environmental problems that had occurred during the past three months. It consisted of four different sections: (1) work environment; (2) work arrangements; (3) allergy history of the employees; and (4) work-related symptoms. The reference data used in FIOH when evaluating the results of this questionnaire are based on the data collected from 122 workplaces in Finland [35,36]. Because of the selected type of the questionnaire as well as the available resources and existing permits, we decided to involve employees and to exclude students from the questionnaire survey.

Employees working throughout the school were requested to participate during the two weeks allotted for responses. The principal of the school was responsible for delivering the questionnaires to the employees, and FIOH collected and reported the answers. Since comparison with the mechanically ventilated section would be misleading, only the questionnaire results from the two hybrid-ventilated building sections are discoursed in this paper.

Potentially significant differences between the two questionnaires were analyzed at Aalto University by SPSS statistical software (SPSS Finland Oy, Espoo, Finland) with a chi-squared test.

## 3. Results and Discussion

### 3.1. Air Flow Rates and Pressure Differences across the Building Envelope

During the initial operation of the ventilation system, the base supply air flow rate in the classrooms was 0 L/s. After inspections and adjustments, the ventilation system was shown to provide sufficient air flow rates. In Classroom 2, the supply air rate was approximately 72–112 L/s (minimum-maximum), and 4.8–7.4 L/s/person when occupancy was estimated to be 15 persons. According to national regulations [37], the minimum requirement is 6 L/s/person, which was met with maximum supply air rate even in the classrooms located most adversely in relation to the air handling unit.

**Figure 5:**
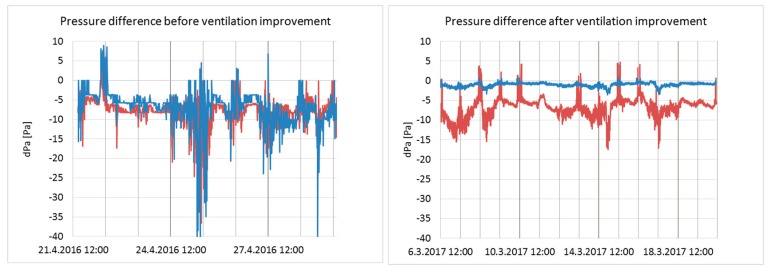
Pressure differences across the envelope in Classrooms 1 (red) and 2 (blue) before and after the ventilation improvement.

Pressure differences across the building envelope in Classrooms 1 and 2 before and after the ventilation improvement are shown in Figure 5.

Before the ventilation improvement, pressure differences varied between −47.1 and 8.9 Pa and between −36.7 and 6 Pa (averages: −7.0 and −7.9 Pa) in Classrooms 1 and 2, respectively. Negative pressures might have enabled infiltration through the building envelope (e.g., from the crawl space or from the wall structures). The pressure differences were more stable after ventilation improvement, especially in Classroom 2, varying between −3.6 and 0.7 Pa (average: −1.0 Pa). Fluctuations decreased also in Classroom 1, but less significantly, remaining in between −17.5 and 4.7 Pa (average: −6.8 Pa). Classroom 1 was the last one in the ventilation supply air corridor, therefore receiving less supply air, which explains higher negative pressure.

In momentary test measurements with several variations of supply air rate and door positions, a positive pressure difference of approximately 6–8 Pa across the envelope occurred in the lobby and in Classrooms 6 and 7 (see Figure 1). Overall, the classroom door positions and classroom-specific supply air rates affected the pressure difference conditions in each room of the building section, thus making the system unstable and difficult to control. This might have caused the variation in the one-week pressure difference measurement results in Classrooms 1 and 2 after ventilation improvement (Figure 5). The occupancy rate and opening of doors and windows in the studied classrooms during measurements are not known.

### 3.2. IAQ Measurements

**Figure 6:**
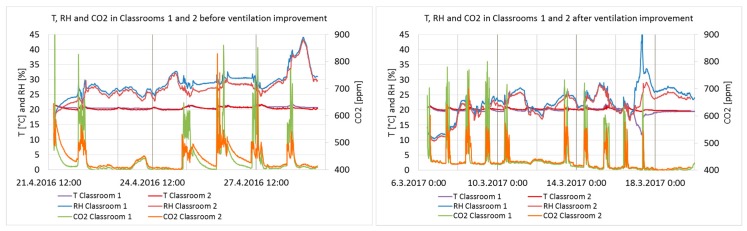
T, RH, and CO_2_ of indoor air in Classrooms 1 and 2 before and after the ventilation improvement.

The T, RH, and CO_2_ of indoor air before and after the ventilation improvement during the entire measurement period are presented in Figure 6.

The T, RH, and CO_2_ of indoor air during school occupancy hours from 8 a.m. to 5 p.m. with directional monthly outdoor temperatures are presented in Table 2. The outdoor data derived from the open access statistics of the Finnish Meteorological Institute, measured at a weather station approximately 6 km from the school. 

The maximum CO_2_ concentrations according to the Finnish Classification are 750 ppm for Category I, 950 ppm for Category II, and 1200 ppm for Category III [38]. The stability of the conditions must be 90% for Categories I and II. Category I is defined as the best possible IAQ, Category II as good IAQ, and Category III as the minimum requirement according to the Finnish regulations. The importance of sufficient ventilation and low CO_2_ levels, especially in school environments, is widely recognized [3]. Lack of base ventilation in the classrooms before the ventilation improvement was not clearly reflected in the CO_2_ concentrations because of the temporary air dilution caused by the CO_2_ sensors. However, when tested by the independent two-sample *t*-test (2-tailed), the difference in CO_2_ concentrations before and after the ventilation improvement is statistically extremely significant at the 100% confidence interval (*p* = 0.000 in Classroom 1, 387 and 740 measurement points; *p* = 0.008 in Classroom 2, 387 and 718 measurement points). Temperatures were stable and at a target level, approximately 21 °C. The meteorological conditions were only slightly differing during the measurement periods.

TVOC concentration was higher before the ventilation improvement (71.5 µg/m^3^) compared to concentration after improvement (10 µg/m^3^); the decrease was 86%. Except for acetone (2 µg/m^3^ before and 7 µg/m^3^ after improvement), single VOC concentrations decreased: decanal and nonanal (2 µg/m^3^ before and 1 µg/m^3^ after improvement), benzaldehyde (2 and 0.9 µg/m^3^), acetic acid (2 and 0 µg/m^3^), and in particular toluene (25 and 6 µg/m^3^) and decamethylcyclopentasiloxane (15 and 0 µg/m^3^). Typical sources for these compounds include building materials, coverings, as well as cleaning and cosmetic products [39,40]. Although TVOC and VOC concentrations were well below the national action values mentioned in the Decree of the Ministry of Social Affairs and Health on health-related conditions of housing and other residential buildings [41], some single VOC concentrations (toluene, decamethylcyclopentasiloxane) measured before the ventilation improvement exceeded the limit values recommended by FIOH [42]. If the recommended limit value is exceeded, it may indicate the existence of an exceptional source and the need for additional environmental investigations [42]. Altogether, the ventilation improvement had a positive effect on VOC concentrations.

The formaldehyde concentration was below 10 ppb (equivalent to approx. 12 µg/m^3^), which is the detection limit of the meter. The PM_2.5_ concentration varied between 0 and 17.6 µg/m^3^ (average 3.8 µg/m^3^), which is below the national limit value of 25 µg/m^3^ [41]. The occupancy rate was low during the measurements because the semester was ending, approximately 15 persons for 1–4 h per day. Since the role of human-based sources was minimal, the measurements present well the building- and ventilation-related conditions.

### 3.3. Characterization of Mycobiota in Indoor Dust

**Figure 7:**
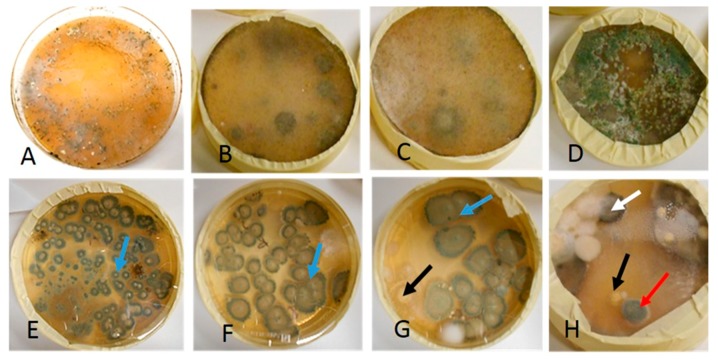
Fungal colonies cultivated from settled dust sampled before (upper row) and after (lower row) the ventilation improvement. Panels (**A**–**C**,**E**–**G**) are cultures from dust samples collected from Classroom 2. Panel (**D**) is a culture from dust collected in the lobby, while Panel (**H**) is a dust culture from Classroom 1. The dust samples were cultivated on MEA and incubated for four weeks at room temperature. The plates in Panels (**A**,**D**) contained over 100 green, *Trichoderma*-like colonies. Colonies in Panel D overgrew the other fungal colonies within two weeks of incubation indicating potential mycoparasitism. The plates in Panels (**B**,**C**) were overgrown with *Rhizopus*-like colonies. The plates in the lower row (Panels (**E**–**H**)) contained mainly green *Penicillium* colonies (blue arrow). The plates in Panels (**G**,**H**) contained yellow *Aspergillus* colonies (black arrow), a black *Aspergillus* colony (white arrow), and a green *Eurotium*/*Aspergillus* colony (red arrow).

Diverse mycobiota cultivated in indoor settled dust was sampled before and after the ventilation improvement and visualized in Figure 7.

The toxic and pathogenic morphotype of the green colonies dominating the plate shown in Panel A of Figure 7 was identified as *Trichoderma citrinoviride*. The rhizoid morphotype in Panels B and C was nontoxic *Rhizopus* sp. unable to grow at 37 °C. The toxic *Trichoderma* sp. colonies in Panel D differed from *T. citrinoviride* due to a lack of ability to grow at 37 °C and association with globous conidia. The dominant morphotypes in Panels E–H were represented by a terverticilliate nontoxic *Penicillium* sp. and a nontoxic monoverticilliate *Penicillium* sp*.* Sporadic toxigenic, black, yellow, and light green colonies in Panels G and H were identified as fungi belonging to *Aspergillus* section *Nigri*, *Aspergillus westerdijkiae*, and *Eurotium* sp., respectively. Fungal colonies representing the dominant morphotypes were tested for toxic and pathogenic potential and identified to the genus or species level as shown in Table 3.

Table 4 shows that the dominant morphotypes cultivated from dust sampled from two locations before the ventilation improvement in May 2016 were the potentially opportunistic human pathogen *T. citrinoviride* [43], toxic, potentially mycoparasitic [44] *Trichoderma* sp., and non-toxic, non-pathogenic *Rhizopus* sp. colonies. The mycobiota cultivated from settled dust collected from three locations after the ventilation improvement in March 2017 was more diverse and characterized by frequent non-toxic *Penicillium* species as well as sporadic toxic and potentially pathogenic *Aspergillus* and *Eurotium* species.

The three most common indoor fungal genera in school environments in continental and moderate climate areas are *Cladosporium* spp., *Penicillium* spp., and *Aspergillus* spp. [45]. In previous studies, *Trichoderma* sp. and *Aspergillus versicolor* have been associated to mold/moisture damage in schools [45].

*Trichoderma citrinoviride* is a potentially opportunistic human pathogen that produces toxic peptaibols and is known to colonize water-damaged buildings [16,17,43]*. Trichoderma longibrachiatum* and *T. harzianum* strains producing peptaibols toxic to mammalian cells have been isolated from water damaged building materials in Finnish dwellings [46]. To our knowledge, this is the first report of the dominant occurrence of potentially pathogenic and allergenic *T. citrinoviride* in indoor settled dust from a Finnish school building. Since settled dust is very likely derived from airborne dust, airborne exposure to viable conidia of *T. citrinoviride* is possible, which is of concern in a school building. Previous studies revealed a significant correlation between the risk of both childhood and adulthood asthma and IgG antibodies to *T. citrinoviride*, suggesting that this species may play a role in the etiology of asthma [47,48]. The cultivated settled dust sampled one year later from the same location after ventilation improvement did not exhibit *T. citrinoviride* colonies. This indicates that the ventilation improvement eradicated the airborne source of viable *T. citrinoviride* conidia.

Viable conidia of toxigenic *Trichoderma* sp. colonies, unable to grow at 37 °C but potentially mycoparasitic, were found before the ventilation improvement in dust sampled from the lobby but were absent in dust sampled from three locations after the ventilation improvement. Many *Trichoderma* species are mycoparasitic [44] and have been reported to indicate moisture and mold growth in buildings [45]. It is possible that the ventilation improvement prevented entry of the *Trichoderma* sp. strains to the indoor environment.

### 3.4. Indoor Air Questionnaire

The indoor air questionnaire results before and after the ventilation improvement from the studied building section (Building Section 1) and from another identical, hybrid-ventilated building section (Building Section 2) are shown in Supplementary Materials (Table S1). According to the information received from the Built Assets Management of the City of Helsinki, equal ventilation improvements occurred in Building Section 1 and Section 2, making the results comparable. Both sections were comprised of two floors with eight classrooms in each floor and with 38–40 employees working in these sections. Classrooms 1 and 2 were located in Building Section 1, as shown in Figure 1. Ventilation improvement was conducted in every classroom of Building Section 1, as well as in Building Section 2. Therefore, the perception responses were requested from the entire staff working in these sections.

The first questionnaire was conducted in May 2016 during the warm spring/summer season, two weeks before the summer holiday began. The second questionnaire was conducted in March 2017 during the cold winter season, halfway through the school semester. It is known that seasonal [49] and psychosocial factors [50,51,52] affect the occupants’ perceptions, making the questionnaire conditions not optimal. The climate conditions of the second questionnaire differed remarkably from those of the first questionnaire and likely affected the responses. The only statistically significant change (at a 50% confidence interval) between the two questionnaires was that the perception of draught increased in both building sections (*p* = 0.04 and *p* = 0.06). Increases in the perception of dry air and too low room temperature were reported as well. These results could be attributed to the cold and dry winter season during the follow-up questionnaire.

In the first questionnaire for Building Section 1, before the ventilation improvement, stuffy air (53%), insufficient ventilation (47%), and unpleasant odor (40%) were most commonly reported, and they had the most significant difference compared to the reference data. After the ventilation improvement, the respondents’ perceptions of these factors decreased to the levels of the reference data. An equivalent but slighter trend in perceptions could be observed in Building Section 2. However, the changes in perceptions before and after the improvement were not statistically significant in either of the building sections. 

An overall increase between the questionnaires could be seen in the perceptions of workplace-related symptoms. The perceptions such as dry skin and throat, cough, and eye irritation were most likely affected by the seasonal and psychosocial factors. In these circumstances, the ventilation improvement did not show any clear effect on the perceived symptoms.

In Building Section 2, the incidence of asthma among the occupants was relatively high, which might have affected the answers, especially in the perceived symptoms. Furthermore, in both building sections, the incidence of hay fever and atopic eczema was high compared to the reference data. 

The number of responses was low for both questionnaires, preventing reliable statistical interpretation of the results. To increase the response rate and to improve the usability of the questionnaires in school environments, the students’ participation via questionnaires applicable for underage individuals [53] is highly recommended.

## 4. Conclusions

This study investigated the functionality of a supply air fan-assisted hybrid ventilation system and the effects of ventilation improvement on measured and perceived IAQ. The study provided several valuable evidences: (1) Major misinterpretation of the ventilation system function is possible if evaluated only by CO_2_ concentration; (2) The studied hybrid ventilation system operating in mechanical supply mode provided adequate ventilation, and was effective in decreasing the concentrations of TVOC and several single VOCs when properly controlled; (3) Microbiological impurities from the building structures to the indoor environment might be prevented with simple adjustments of ventilation, once proper supply air income is being ensured via the ventilation system and negative pressure differences are being controlled; (4) Ventilation improvements might decrease the perceptions of unpleasant odors and stuffy air; (5) A small number of respondents and seasonal effects on the perceptions of the participants weakened the ability to interpret the results of the questionnaire in relation to ventilation operation. The usability of a questionnaire in an intervention study could be improved by conducting questionnaires during the same season and by optimizing the number of respondents by including students in the survey.

The findings of this study could be utilized for the evaluation of the usability and effect of hybrid ventilation systems on IAQ in school buildings or for seeking solutions in reducing exposure to indoor-generated pollutants in buildings with IAQ complaints and occurred symptoms. However, these findings should be confirmed with further studies that consider several school buildings with different ventilation types and with extended questionnaire surveys that include student participation.

## Figures and Tables

**Table 1:** Measurement methods, devices, and their accuracy, measurement place, and duration.

Measured Factor	Device	Accuracy	Place	Time
Supply air flow rate	SWEMA 3000 md	±0.3% read value, lowest ±0.3 Pa	All classrooms	60 s average
Pressure difference across the envelope	KIMO CP101, logger Grant 1000	1.5% of reading ±3 Pa	Classrooms 1 and 2	Continuous, 1 week (May 2016)
	Envic dp-101s-pd2, logger Grant 1000	3% of reading ±0.2 m/s	Classrooms 1 and 2	Continuous, 2 weeks (March 2017)
Temperature (T)	Rotronic CL11	±0.3 °C	Classrooms 1 and 2	Continuous (1–2 weeks)
Relative humidity (RH)	Rotronic CL11	±3% (10 ... 95%)	Classrooms 1 and 2	Continuous (1–2 weeks)
Carbon dioxide (CO_2_)	Rotronic CL11	±(30 ppm + 5% of reading)	Classrooms 1 and 2	Continuous (1–2 weeks)
Formaldehyde	FM-801	±10 ppb at 40, 80, 160 ppb	Classroom 2	Continuous, 1 week
Particulate matter 2.5 μm (PM_2.5_)	MIE pDR-1500	±5%	Classroom 2	Continuous, 1 week
Volatile organic compounds (VOCs)	Tenax TA, TD-GC-MS	±20% (average)	Classroom 2	40 min
Mycobiota of settled dust	Plastic bag, MEA plates, BSMI, ICP, ITS		Classrooms 1 and 2, lobby	Cultivated for 4 weeks
Perceived indoor air quality	Örebro (MM40)-questionnaire (Finnish Institute of Occupational Health (FIOH))		Personnel of the whole building	2-week response time

**Table 2:** Minimum, maximum and average values of RH, T, and CO_2_ in Classrooms 1 and 2, during 8 days before and 14 days after the ventilation improvement during school occupancy hours from 8 a.m. to 5 p.m., and outdoor temperatures per month in question.

		Classroom 1			Classroom 2			Outdoor, Per Month *
		RH (%)	T (°C)	CO_2_ (ppm)	RH (%)	T (°C)	CO_2_ (ppm)	T (°C)
Before	Min	18	20	394	16	20	402	−1.4
	Max	40	22	1431	38	22	829	13.7
	Average	29	21	488	27	21	458	4.9
After	Min	11	12	394	10	20	400	−7.8
	Max	46	22	801	29	21	700	11.9
	Average	23	20	464	22	20	450	1.0

* Finnish Meteorological Institute.

**Table 3:** The eight fungal morphotypes isolated from settled dust collected before and after the ventilation improvement, characterized by toxigenicity, pathogenic potential, and conidiophore morphology.

		Toxicity		Colony Color	Size of Conidia/Spores	Morphology under Light Microscope
	Growth at 37 °C	BSMI	ICP	MEA	(μm)	
*Aspergillus* section *Nigri* 1 strain	+	-	+	Black	3.5–5	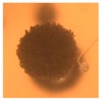
*Asp. westerdijkiae* 2 strains	-	+	+	Yellow	2.5–3	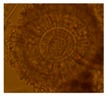
*Eurotium *sp*.* 1 strain	+	+	+	Green	5–7	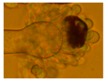
*Penicillium *sp. 10 strains (Terverticilliate)				Green	3.4	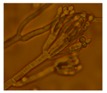
*Penicillium *sp. 3 strains (Monoverticilliate)				Green	2.3	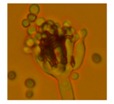
*Rhizopus *sp*.* 10 strains	-	-	-	Grey	5–10	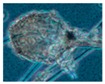
Trichoderma citrinoviride * 10 strains	+	+	+	Green	1.6 × 3	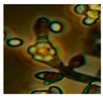
*Trichoderma *sp. 5 strains	-	+	+	Green	4	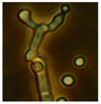

* Identified to species level by ITS sequence analysis.

**Table 4:** Cultivable mycobiota in settled indoor dust sampled from surfaces above floor level in four different locations of the school. Dust was sampled before and after the ventilation improvement and cultivated on three plates per location.

School Samples	Settled Dust		
Before ventilation improvement	Sampled 31 May 2016	Number of colonies/plate	Number of plates containing a colony morphotype/all plates
Locations: Classroom 2 and lobby	*Trichoderma citrinoviride* ^a,b^	>100	1/6
	*Rhizopus* sp.	Plate overgrown	2/6
	*Trichoderma* sp. ^a^	>100	2/6
After ventilation improvement	Sampled 6 March 2017		
Locations: Classrooms 1, 2 and 7	*Penicillium* sp. ^c^ *Penicillium* sp. ^d^	>100–120 10	3/9 2/9
	*Aspergillus westerdijkiae* ^a^	2–3	2/9
	*Asp. Niger* ^a,b^	1–2	3/9
	*Eurotium* sp. ^a^	1	1/9

^a^ Colonies of morphotypes that are toxic to sperm or kidney cells; ^b^ Colonies of potentially pathogenic morphotypes able to grow at 37 °C; ^c^ Terverticilliate *Penicillium* species; ^d^ Monoverticilliate *Penicillium* species.

## Supplementary Materials

The following are available online at http://www.mdpi.com/1660-4601/15/7/1414/s1, Table S1: Indoor air questionnaire (Finnish Institute of Occupational Health© 2006–2008, version 2.0) results from May 2016 and January 2017.

## Abbreviations

The following abbreviations are used in this manuscript:

IAQ	Indoor air quality
TVOC	Total volatile organic compounds
PM_2.5_	Fine particulate matter, particle size 2.5 μm
RH	Relative humidity
VOC	Volatile organic compounds
T	Temperature
CO_2_	Carbon dioxide
FIOH	Finnish Institute of Occupational Health
MEA	Malt extract agar
BSMI	Boar sperm motility inhibition
ICP	Inhibition of cell proliferation
ITS	Internal transcribed spacer
