# Supplementary material for: Effects of Ventilation Improvement on Measured and Perceived Indoor Air Quality in a School Building with a Hybrid Ventilation System
Source: Int J Environ Res Public Health. 2018 Jul 5;15(7):1414. doi: 10.3390/ijerph15071414 (PMC6068750; doi:10.3390/ijerph15071414)
Supplement: Supplementary file 1 [file ijerph-15-01414-s001.pdf]

Table S1. Indoor air questionnaire (Finnish Institute of Occupational Health© 2006–2008, version 2.0) results from May 2016 and January 2017.

	Reference data	Building Section 1		*p*-Value	Building Section 2		*p*-Value
	[1-3]	2016	2017		2016	2017	
**Background information**							
Number of answers		15	16		16	17	
Response rate (%)	71	79	80		84	85	
Females (%)	21	87	88		94	94	
Daily smokers (%)		13	6		0	0	
Average age (years)		41	42		41	38	
Average employment in this work place (years)		5	5		4	4	
**Work environment (%)**		%	%				
Draught	22	7	44	**0.04***	13	47	**0.06***
Room temperature too high	17	0	0	-	0	6	1.00*
Varying temperature	16	20	31	0.69*	19	27	0.69*
Room temperature too low	13	27	56	**0.10**	19	53	**0.04**
Stuffy air	34	53	38	0.38	80	71	0.69*
Dry air	35	13	44	0.11*	14	41	0.13*
Insufficient ventilation	32	47	31	0.38	75	59	0.33
Smell of mold	9	7	0	0.48*	7	6	1.00*
Unpleasant odour	17	40	19	0.25*	38	24	0.47*
Environmental tobacco smoke	4	0	0	-	13	0	0.23*
Noise	17	47	56	0.59	19	50	**0.06**
Dim light or reflections	14	7	13	1.00*	6	0	0.49*
Dust or dirt	25	27	25	1.00*	25	35	0.71*
**Work regarded as interesting and stimulating (%)**							
Often	75	73	88	0.39*	88	82	1.00*
Sometimes	20	27	13		13	18	
Seldom or never	4	0	0		0	0	
**Too much work to do (%)**							
Often	20	0	13	**0.08***	13	18	0.70*
Sometimes	59	40	63		56	65	
Seldom or never	21	60	25		31	18	
**Opportunity to influence work conditions (%)**							
Often	35	27	25	0.51*	25	24	0.58*
Sometimes	44	60	75		63	47	
Seldom or never	21	13	0		13	29	
**Fellow workers help with problems in the work (%)**							
Often	72	87	88	1.00*	88	76	**0.05***
Sometimes	22	13	13		0	24	
Seldom or never	6	0	0		13	0	
**Allergic diseases (%)**							
Asthma	8	0	0	-	19	18	1.00*
Hay fever	38	67	56	0.55*	50	41	0.61
Atopic eczema	28	40	19	0.25*	13	12	1.00*
**Stress (%)**							
Very much	10	7	0	0.45*	13	24	0.74*
Some	28	27	47		50	35	
None/only a little	63	67	53		38	41	
**Symptoms (%)**							
Fatigue	16	7	19	0.60*	19	29	0.69*
Heavy-headedness	9	20	13	0.65*	6	35	**0.09***
Headache	7	13	6	0.60*	19	29	0.69*
Difficulty concentrating	3	0	6	1.00*	6	18	0.60*
Eye irritation	17	27	31	1.00*	20	41	0.27*
Irritated, stuffy, or running nose	20	13	25	0.65*	19	35	0.44*
Hoarse/dry throat	14	13	38	0.22*	31	35	0.81
Cough	5	0	13	0.48*	6	18	0.60*
Cough disturbing sleep	1	0	6	1.00*	0	0	-
Dry or flushed facial skin	11	7	25	0.33*	13	25	0.65*
Hands: dry, itching, red skin	15	0	19	0.23*	7	24	0.34*
Shortness of breath	3	7	0	0.48*	0	6	1.00*
Wheezing	1	7	0	0.48*	0	6	1.00*
Fever or chills	2	0	0	-	7	6	1.00*
Joint pain	3	0	0	-	0	0	-
Muscular pain	4	0	0	-	0	0	-
Other		0	0	-	9	12	0.83*

Statistically significant changes at a 10% confidence interval ($p < 0.1$) are bolded. The p-values marked with * were determined by the Fisher's exact test (SPSS). Reference data are based on analysis of the comprehensive questionnaire data collected by FIOH.
